# Intranasal cryotherapy for refractory chronic rhinitis: a prospective study

**DOI:** 10.1017/S0022215125104258

**Published:** 2026-04

**Authors:** Charlotte Arnold, Simon Morris, Owen Bodger, Heikki Whittet

**Affiliations:** 1 Department of ENT, Morriston Hospital, Swansea Bay University Health Board, Swansea, Wales, UK; 2 Department of Healthcare NHS Trust Medical Education, Imperial College London, London, UK; 3 Department of Health Data Science, Swansea University, Swansea, Wales

**Keywords:** rhinitis, cryotherapy, Sino-Nasal Outcome Test, nasal obstruction, United Kingdom

## Abstract

**Objective:**

To assess the efficacy of intranasal cryotherapy to treat chronic rhinitis refractory to medical therapy.

**Methods:**

An evaluation was performed for all patients (*n* = 36) with chronic rhinitis refractory to medical treatment who underwent intranasal cryotherapy between 2022 and 2024 at this centre. The primary outcome measures were changes in validated pre- and post-operative scoring systems (Total Nasal Symptom Score, Sino-Nasal Outcome Test 22 (SNOT-22), Nasal Obstruction Symptom Evaluation and peak inspiratory nasal flow).

**Results:**

Objective scoring pre- and post-procedure showed statistically significant improvement across all measures (*p* < 0.001): mean Total Nasal Symptom Score (12 hours) 8.4 to 5.3, Total Nasal Symptom Score (2 weeks) 9.2 to 5.9, SNOT-22 56 to 31, Nasal Obstruction Symptom Evaluation 57.5 to 28.5 and peak inspiratory nasal flow 98 to 138 l/min.

**Conclusion:**

This is the only dataset for patients receiving intranasal cryotherapy in the UK to date and follows patients over a two-year period. The results support the ongoing use of intranasal cryotherapy for sustained treatment of refractory chronic rhinitis.

## Introduction

Chronic rhinitis is a common condition, with 26 per cent of the UK adult population estimated to be affected.^1^ The mainstays of treatment include allergen avoidance, nasal douching, topical intra-nasal steroids, anti-cholinergics or decongestant therapy to control the hallmark symptoms of nasal obstruction, congestion and discharge.^2^

Those patients refractory to medical management may be offered surgical intervention in the form of inferior turbinate surgery, vidian neurectomy or posterior nasal nerve sectioning.^3^^,^^4^ Whilst the latter treatments have positive long-term outcomes, they are typically performed under general anaesthetic and have high complication rates, including dry eyes, visual changes, numbness and facial pain.^5^^,^^6^

An emerging technique to target the posterior nasal nerve is cryosurgical ablation. This aims to reduce the parasympathetic tone and response to nasal antigens, thus providing symptomatic relief from nasal hypersensitivity in chronic rhinitis.^7^ Cryoablation potentially offers a less invasive option than posterior nasal nerve sectioning and vidian neurectomy.

The Stryker ClariFix device is a hand-held, endoscope-compatible instrument that releases a nitrous oxide cryogen from the instrument tip.^7^ This freezes the target mucosa and cryoablates the posterior nasal nerve at the level of the inferior turbinate. Thus far, a number of small studies have shown reductions in nasal symptoms, but there is uncertainty regarding this as a long-term management option that can reduce the burden of disease, reduce steroid dependence and increase discharge rates from ENT clinic in the UK setting.^8^^–^^10^

The National Institute for Health and Care Excellence (NICE) has licenced the use of the Stryker ClariFix device for use in the UK under the condition that it is monitored prospectively within a study setting, pending further evidence review for wider dissemination.^8^

## Materials and methods

### Aims

This study aimed to assess the efficacy of intranasal cryotherapy when used to treat chronic rhinitis refractory to medical therapy.

### Design

Prospective evaluation was performed for all patients undergoing intranasal cryotherapy between March 2022 and July 2024 (28 months) in a single regional ENT unit. This was performed as part of a service evaluation to assess the effectiveness of intranasal cryotherapy at our site. Data were collected on patient demographics, current medical management, co-morbidity and indication for intervention.

The main outcome measures were pre-and post-operative patient recorded outcome measures. Patient recorded outcome measures were measured using three validated subjective scoring systems (Total Nasal Symptom Score, Sino-Nasal Outcome Test (SNOT-22) and Nasal Obstruction Symptom Evaluation) and objective peak inspiratory nasal flow (peak inspiratory nasal flow, l/min). Post-operative results were taken at their first clinic visit (mean, 2.67 months; range, 1–12 months). Chart review at subsequent follow up determined secondary outcome measures.

Secondary outcomes included whether patients required continued medical therapy or suffered adverse events and length of follow up.

### Participants and setting

Patients were identified prospectively, with data collated from operative records, electronic and paper files. All 36 patients underwent the procedure under local anaesthesia in the out-patient department at Morriston Hospital, Swansea between 2022 and 2024. Data collection was completed in 2025 to permit minimum 12 months follow-up data for all patients. Sample size was determined by logistical constraints and limited by the number of procedures performed at this unit during this timeframe.

The authors assert that all procedures contributing to this work comply with the ethical standards of the relevant national and institutional guidelines on human experimentation (Medicines and Healthcare products Regulatory Agency) and with the Helsinki Declaration of 1975, as revised in 2008. Formal ethical approval was not required based on the National Health Service Health Research Authority tool. Approval was granted to begin offering ClariFix cryotherapy by the health board in February 2022 under a service evaluation procedure with prospective evaluation. Approval was granted by the hospital’s Joint Study Review Committee on 5 June 2024 for collection, dissemination and publication of results.

#### Inclusion criteria

All patients over 18 years of age undergoing intranasal cryoablation for chronic rhinitis, who had failed trial of optimal medical management and where no confounding mechanical obstruction or alternative pathology accounted for presenting symptoms were included.

#### Exclusion criteria

Patients undergoing intranasal cryotherapy as part of wider procedure, including septoplasty, concha bullosa reduction or nasal valve surgery, were excluded.

### Operating procedure

Standard practice in this unit is for patients to be assessed by one consultant rhinologist, where baseline endoscopic examination and patient-reported outcome measures are collected. Patients identified to have chronic rhinitis with no other confounding explanation or correctable mechanical deformity are trialled on three months of optimal medical management including saline nasal douching, topical nasal steroid, and anti-cholinergic and anti-histamine preparations. If symptoms persist, patients are offered intervention in a local anaesthetic out-patient setting through either intranasal cryotherapy alone (Stryker ClariFix device) or in combination with inferior turbinate reduction (using a Coblation needle). The decision to offer concurrent inferior turbinate reduction is based on obstructive symptom burden or perceived size and reactivity of hypertrophied inferior turbinates on clinical assessment. It is noted that 18 out of the 36 patients included underwent concurrent inferior turbinate reduction with a Coblation needle. The outcomes for these two treatment groups were compared as part of our analysis.

Follow up is initially offered at four to six weeks post-operatively, where initial post-patient recorded outcome measures are collected. Patients then enter a patient-initiated follow-up regimen where they can request to be assessed again. Hence subsequent follow up is not standardised across all participants.

### Statistical methods

Data were collected and stored anonymously on Microsoft Excel. IBM SPSS (Armonk, NY, United States) was used to complete statistical analysis. Statistical analysis aimed to assess the following research questions: (1) pre- and post-procedure comparison of primary outcome measures; (2) what impacts outcomes and do age, sex, co-morbidities or concurrent turbinate reduction affect outcomes; (3) comparison of outcomes in those who underwent cryotherapy alone versus cryotherapy in combination with inferior turbinate reduction; and (4) does timing of follow up impact outcomes?

The outcomes metrics (Total Nasal Symptom Score, SNOT, Nasal Obstruction Symptom Evaluation, peak inspiratory nasal flow) were all measured on a continuous scale, bounded above and below, ensuring good convergence properties. For this reason, parametric methods were deemed sufficiently robust despite the modest sample size. All data were pre- and post-operative values and so a repeated measures design was chosen and the analysis performed using a Repeated Measures Analysis of Variance. Simple pre- and post-comparisons were performed without correction for other variables.

When assessing the impact of patient characteristics and concurrent procedures, an appropriate covariate was added to the model. The results, assessing correlation between scores and time to follow up, were generated using Pearson correlations and partial correlations where a correction for pre-operative score was required.

Wherever a hypothesis test was performed the *p* value was reported; the effect size and confidence interval were only reported where it was felt that the effect size was significant, with a 5 per cent level of significance used throughout. Power calculations were not performed because this was a prospective service evaluation with multiple components.

## Results and analysis

### Participants

Of the 36 patients identified for inclusion in the study, the effective sample size stated varied because of incomplete data sets. Table 1 gives the patient demographics of the cohort, the mean age of participants was 58 years, with a 61 to 39 per cent male to female ratio. Indications for cryotherapy included chronic secretomotor rhinitis (*n* = 34), allergic seasonal rhinitis (*n* = 1) and gustatory rhinitis (*n* = 1). It was found that 41.7 per cent (*n* = 15) of patients had co-morbid atopic disease, including eczema, asthma and hayfever.

**Table 1.** Patient demographics*

Characteristic	Value
Sex (*n* (%))	
– Male	22 (61)
– Female	14 (39)
Age (median (IQR); years)	58 (20−87)
Pre-operative nasal steroid use (*n* (%))	
– Yes	35 (97)
– Unknown	1 (3)
Relevant co-morbidity (*N* = 18) (*n* (%))	
– Hayfever	11 (31)
– Asthma	4 (11)
– Other^†^	3 (8)
Concurrent procedure under local anaesthetic (*n* (%))	
– Inferior turbinate reduction	18 (50)
– None	18 (50)

* Total *N* = 36.

† Other comorbidities included: fibromyalgia, house dust mite allergy and rheumatoid arthritis. IQR = interquartile range.

### Main outcome measures

The primary objective was to determine the efficacy of intranasal cryotherapy by comparing pre- and post-operative scores across a range of subjective and objective domains. Summaries of the RMANOVA scores can be found in Table 2.

**Table 2.** Summary figures for pre- and post-operative patient scores

Measure of nasal symptoms	Pre-operative (mean ± SD)	Pre-operative 95% CI	Post-operative (mean ± SD)	Post-operative 95% CI	*p* value	Effect size (95% CI)	Eta-square	Percentage improving (%)
Total Nasal Symptom Score at 12 hours	8.4 (± 3.0)	7.4−9.4	5.3 (± 3.5)	4.0−6.7	<0.001	−3.3 (−4.8, −1.8)	0.438	79
Total Nasal Symptom Score at 2 weeks	9.2 (± 3.2)	8.1−10.2	5.9 (± 4.3)	4.3−7.6	<0.001	−3.5 (−5.1, −1.9)	0.438	79
SNOT-22	56.0 (± 23.6)	48.0−64.0	31.1 (± 20.3)	23.2−39.0	<0.001	−25.6 (−36.0, −15.3)	0.488	82
Nasal Obstruction Symptom Evaluation	57.7 (± 30.2)	47.5−67.9	28.5 (± 24.9)	18.6−38.4	<0.001	−31.1 (−43.9, −18.3)	0.487	78
Peak inspiratory nasal flow	98.0 (± 40.5)	84.1−111.9	138.7 (± 50.8)	118.2−159.2	<0.001	+44.3 (30.6, 58.2)	0.638	96

SD = standard deviation; CI = confidence interval; SNOT-22 = Sino-Nasal Outcome Test 22.

There is a statistically significant improvement in the mean scores across all objective and subjective measures at the 0.1 per cent level of significance. The mean Total Nasal Symptom Score (12 hours) improved from 8.4 to 5.3, Total Nasal Symptom Score (2 weeks) improved from 9.2 to 5.9, SNOT22 improved from 56 to 31, Nasal Obstruction Symptom Evaluation improved from 57.5 to 28.5 and peak inspiratory nasal flow improved from 98 to 138 l/min. The impact of treatment on patients was favourable, as demonstrated by the ‘large’ effect size of the eta-square results. In addition, the scores are positive on an individual level, with 79–82 per cent of patients seeing an improvement in their patient recorded outcome measures and 96 per cent demonstrating improved peak inspiratory nasal flow.

### Patient outcome and follow up

Initial clinical follow up was standardised at four to six weeks post-operatively, with decision to discharge or offer patient-initiated follow up thereafter. The mean duration of follow up was 8.4 months (± 6.7 standard deviation) with a range between 1 and 24 months.

Many patients (50 per cent, *n* = 18) were discharged after first post-operative review. A total of 50 per cent (*n* = 18) of patients continued to use some form of medical therapy post-operatively, with 36 per cent (*n* = 13) of patients using nasal steroids post-operatively. Of those who were discharged, 44 per cent (*n* = 8) did not require use of nasal steroids.

### Adverse events

Three patients (8.3 per cent) experienced adverse events: maxillary discomfort, unilateral paraesthesia (upper lip, teeth and cheek) and epistaxis. No major adverse events were reported.

### What impacts outcomes?

Further Repeated Measures Analysis of Variance (RMANOVA) analysis was performed to identify whether intranasal cryotherapy success was influenced by patient characteristics and performance of concurrent inferior turbinate reduction (Table 3). The only factors which show any association with the scores are the presence of concurrent inferior turbinate reduction for Total Nasal Symptom Score (12 hours) (*p* = 0.044) and patient sex for peak inspiratory nasal flow (*p* = 0.031). The effect of patient sex only seems to apply to the peak inspiratory nasal flow score and sees female patients respond more than male patients. There was no measurable effect related to age or co-morbidity. Essentially, the addition of inferior turbinate reduction was not statistically significantly superior in this cohort.

**Table 3.** Impact of independent variables on the outcome of surgery patient

Independent variable	Sex (*p* value)	Age (*p* value)	Co-morbidities (*p* value)	Concurrent inferior turbinate reduction (*p* value)
Comparison (post *vs* pre)				
Total Nasal Symptom Score (12 hours)	0.407	0.717	0.771	0.044*
Total Nasal Symptom Score (2 weeks)	0.470	0.896	0.964	0.106
SNOT-22	0.273	0.613	0.880	0.057
Nasal Obstruction Symptom Evaluation	0.374	0.639	0.881	0.090
Peak inspiratory nasal flow	0.031*	0.168	0.596	0.680

* *p* value <0.05. While sex, the presence of co-morbidities and concurrent inferior turbinate reduction are binary factors, age is included as a continuous covariate. SNOT-22= Sino-Nasal Outcome Test 22.

### Comparison of concurrent inferior turbinate reduction versus cryotherapy alone

**Figure 1.**
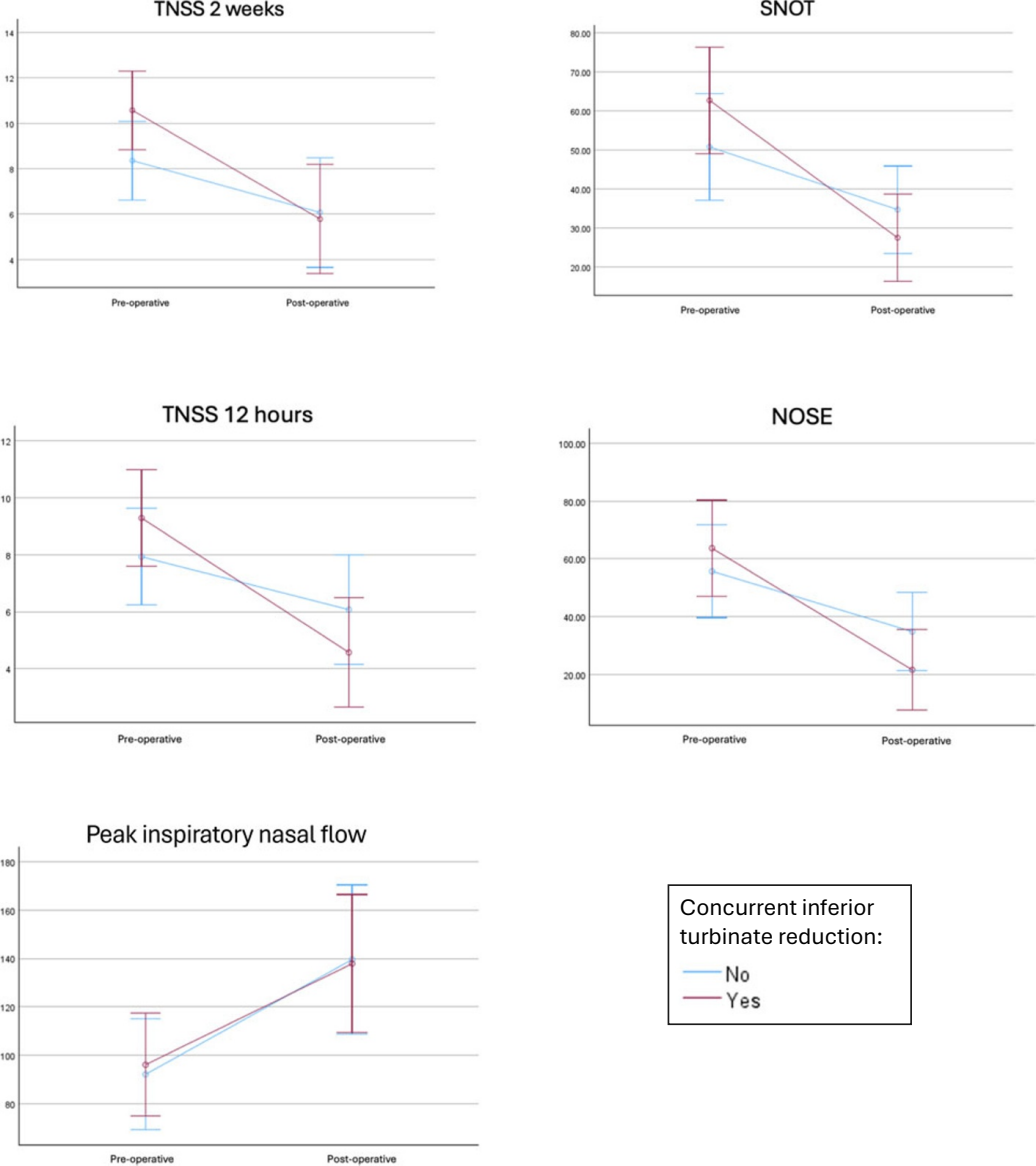
Comparison of main outcomes for patients undergoing cryotherapy alone versus concurrent inferior turbinate reduction. Error bars represent 95 per cent confidence intervals. TNSS 2 weeks = Total Nasal Symptom Score at 2 weeks; SNOT-22 = Sino-Nasal Outcome Test 22; TNSS 12 hours = Total Nasal Symptom Score at 12 hours; NOSE = Nasal Obstruction Symptom Evaluation.

In this study sample, 50 per cent of patients (*n* = 18) were offered concurrent inferior turbinate reduction surgery with cryotherapy. When assessing these groups independently, those who were offered concurrent inferior turbinate reduction had more severe baseline patient-recorded outcome measures when compared with the cryotherapy alone group. However, the effect size was greater in those undergoing concurrent cryotherapy and inferior turbinate reduction, as demonstrated in Figure 1 and Table 4.

**Table 4.** Comparison of outcomes with cryotherapy alone versus cryotherapy with inferior turbinate reduction

Comparison (post vs pre)	*p* value	Effect size (95% CI)	Eta-square	Percentage improving (%)
Cryotherapy alone				
– Total Nasal Symptom Score 12 hours	0.013	−1.9 (−3.25, −0.49)	0.389	71
– Total Nasal Symptom Score 2 weeks	0.021	−2.3 (−4.17, −0.40)	0.345	71
– SNOT-22	0.005	−16.1 (−26.42, −5.72)	0.464	86
– Nasal Obstruction Symptom Evaluation	0.017	−20.9 (−37.41, −4.30)	0.363	71
– Peak inspiratory nasal flow	0.002	+47.5 (22.25, 72.75)	0.609	100
Cryotherapy + inferior turbinate reduction				
– Total Nasal Symptom Score 12 hours	0.002	−4.7 (−7.27, −2.16)	0.891	86
– Total Nasal Symptom Score 2 weeks	0.002	−4.8 (−7.40, −2.17)	0.546	86
– SNOT-22	<0.001	−35.2 (−53.18, −17.25)	0.580	79
– Nasal Obstruction Symptom Evaluation	<0.001	−42.1 (−62.49, −21.66)	0.627	85
– Peak inspiratory nasal flow	<0.001	+41.8 (24.55, 59.02)	0.879	93

SNOT-22 = Sino-Nasal Outcome Test 22.

The mean effect size in the cryotherapy-alone group was −1.86 in the Total Nasal Symptom Score (12 hours), −2.29 in the Total Nasal Symptom Score (2 weeks), −16.07 in SNOT-22 and −20.86 in the Nasal Obstruction Symptom Evaluation. Conversely, in the concurrent inferior turbinate reduction group, scores were −4.7, −4.79, −35.2 and −42.08, respectively. Despite the difference in effect size seen between the two groups, based on this sample there was no statistical difference with addition of inferior turbinate reduction to the cryotherapy procedure, except for Total Nasal Symptom Score scores at 12 hours post procedure. However, the patients in the inferior turbinate reduction group have a higher symptom burden based on their pre-operative patient-recorded outcome measures, which may confound results.

For the objective peak inspiratory nasal flow scores, no difference was observed between the groups in terms of pre-scores, post-scores or average improvement. The mean effect size of cryotherapy alone on peak inspiratory nasal flow was +47.5 and +41.8 l/min for the concurrent inferior turbinate reduction group.

### Does timing of post-operative measurements affect outcomes?

The median follow up in this cohort was 6 months, with a mean of 8 months (range, 1–24 months). In those patients that had follow up after their initial four-to-six-week appointment, subsequent patient-recorded outcome measures and peak inspiratory nasal flow were recorded at each stage. As such, many patients have long-term follow-up results, from which we can assess the longevity of intervention. Pearson correlations were calculated to look for correlations between post-operative outcomes measures and time delay. The results in Table 5 show that there was no correlation between post-operative scores over time (*p* < 0.05), improvement in score over time or post-operative scores corrected for severity of symptoms.

**Table 5.** Significance of correlations between patient reported outcome measure scores and change in scores, with the time to follow up

Score	Pre-operative score *vs* time	Post-operative score *vs* time	Improvement *vs* time	Post score *vs* time (corrected for pre score)
Total Nasal Symptom Score 12 hours	0.225	0.109	0.738	0.232
Total Nasal Symptom Score 2 weeks	0.096	0.404	0.503	0.970
SNOT-22	0.554	0.892	0.416	0.693
Nasal Obstruction Symptom Evaluation	0.143	0.718	0.256	0.514
Peak inspiratory nasal flow	0.007*	0.332	0.662	0.654

* Statistical significance <0.01. SNOT-22 = Sino-Nasal Outcome Test 22.

## Discussion

Medical management of chronic rhinitis typically involves daily use of topical preparations and may still be incomplete in controlling patients’ symptoms.^11^^,^^12^ Intranasal cryotherapy offers a novel, minimally invasive approach to refractory chronic rhinitis, which can be performed in a clinic setting under local anaesthetic. Our study demonstrates a strong safety profile of the procedure with minimal adverse events, in keeping with the current literature.^13^

This study demonstrates improvement across a range of disease-specific quality of life measures in patients undergoing cryoablation for chronic rhinitis. The patient data captured reflect the typical spectrum of difficult to manage, refractory chronic rhinitis symptoms and co-morbidities, including atopic disease. Overall, there is significant improvement in three validated scoring systems and objective testing through peak inspiratory nasal flow measurements, which presents a more comprehensive assessment of patient-recorded outcome measures than in current published literature, which primarily use Total Nasal Symptom Score alone.^10^^,^^11^^,^^14^

In addition, when comparing our data to the current literature, our patients have higher baseline Total Nasal Symptom Scores (mean, 8.4 (12 hours) to 9.2 (2 weeks), compared with those between 6 and 7 in recent meta-analyses), suggesting our patient group has more severe rhinitis.^15^ Despite this, results demonstrate improvement in Total Nasal Symptom Score in 79 per cent of patients assessed; a higher level of improvement than the 71 per cent reported in Choi *et al*.’s recent systematic review.^15^ In addition, this is the first prospective study, to our knowledge, to use peak inspiratory nasal flow to evaluate response to nasal obstruction using cryotherapy, with 96 per cent of patients experiencing improvement in nasal airflow.

A potential limitation of intranasal cryotherapy is its long-term outcomes.^8^ In this study, patients were followed up for a mean of 8 months and no decay in post-operative results was observed over time (for means in all outcome measures), even in cases followed up for up to 24 months post-operatively. In addition, 50 per cent of patients were successfully discharged after initial follow up. This affirms the outcomes of cryoablation when compared with Young *et al*., whose follow up concluded at three months post-operative.^14^

Previous criticisms have suggested that the cost of an intranasal cryotherapy device does not outweigh the ongoing disease burden and need for topical nasal steroids. The ClariFix and turbinate Coblation wands cost our centre £960 and £90, respectively, whereas depending on the nasal spray used, topical medications could cost between £77 a year (fluticasone) and £300 a year (ipratropium bromide), so cost is still a consideration.^16^

However, in this study the number of patients requiring nasal steroids reduced from 97 to 36 per cent, which does indicate a benefit of the procedure in reducing topical steroid dependency. These results supersede previous findings by Rosi-Schumacher *et al*., who reported 28.6 per cent of patients discontinuing medications.^17^

Significance was identified in those who underwent intranasal cryotherapy alone and those who underwent concurrent inferior turbinate reduction. Indeed, a combination may be more beneficial in those with the most severe symptoms. As outcomes were analysed prospectively, it emerged that an improvement in obstructive symptoms was a surprising yet beneficial aspect of this treatment modality, with 100 per cent of patients undergoing cryotherapy alone having improved mean peak inspiratory nasal flow scores by 47.5 l/min. Furthermore, combined cryotherapy with inferior turbinate reduction resulted in a 42.0 l/min increase in peak inspiratory nasal flow. Thus, obstruction may be an additional prime indicator for cryotherapy alone. As such, it is essential that an assessment is made of the patient’s symptom load before intervention is offered.

- Chronic rhinitis is a common condition with a high burden of disease
- Intranasal cryotherapy offers a minimally invasive surgical option to cryoablate the posterior nasal nerve to provide symptomatic relief
- This is the first dataset for intranasal cryotherapy of patients in the UK
- Intranasal cryotherapy is a safe procedure, suitable to be performed under local anaesthetic in the out-patient setting
- Sustained responses were seen in all primary outcome measures for those suffering with refractory chronic rhinitis

### Limitations and strengths

Previous criticism of intranasal cryotherapy in NICE guidance has drawn on the length of the sustained response.^8^ This is the first study of intranasal cryotherapy in the UK to date and the first to report peak inspiratory nasal flow measurements worldwide. We believe these data demonstrate safety, significant efficacy and longevity of benefit, but a further long-term prospective study is required to determine the long-term effects of this intervention.

The limitations of our study include a relatively small sample size subject to type II error and loss of six patients to follow up. The authors also acknowledge that patients undergoing concurrent inferior turbinate reduction introduce a potential confounder, but those who underwent cryotherapy alone still had significant improvements in symptoms. Whilst the subjective scoring measurements used, such as the Total Nasal Symptom Score, may be subject to recall bias, the objective scores used in this study are more robust. In future, a high-powered randomised control study would need to be performed to affirm the findings of this prospective study, with comparison of interventions and subgroup analysis according to primary symptom burden and rhinitis sub-type.

## Conclusion

Our data support the ongoing use of intranasal cryotherapy for sustained treatment of refractory chronic rhinitis in the UK. It can be performed in the out-patient setting, avoiding the requirement for general anaesthetic, as well as potentially reducing the need for ongoing topical nasal medical therapy and increasing discharge rates from ENT clinics. Future randomised studies would be beneficial in supporting wider use of the cryoablation in chronic rhinitis.

## Data Availability

Data are available on request from the authors.
